# Telerehabilitation and Face-to-Face Exergame Delivery Modalities to Improve Postural Control in Children with Cerebral Palsy: A Randomised Controlled Trial

**DOI:** 10.3390/medsci14020246

**Published:** 2026-05-08

**Authors:** Valeska Gatica-Rojas, L. Eduardo Cofré Lizama

**Affiliations:** 1 Human Motor Control Laboratory, Faculty of Health Sciences, Universidad de Talca, Av. Lircay S/N, Talca 3460000, Chile; 2 Department of Allied Health, School of Health Sciences, Swinburne University of Technology, Melbourne, VIC 3122, Australia; eduardocofre@swin.edu.au; 3 Department of Medicine (Royal Melbourne Hospital), The University of Melbourne, Melbourne, VIC 3052, Australia

**Keywords:** telerehabilitation, children, cerebral palsy, postural control, balance control, virtual reality, exergame, physical therapy

## Abstract

**Background:** Low-cost virtual reality exergames may help maintain and improve postural control in children with spastic hemiplegia cerebral palsy (CP). This study aimed to evaluate the comparative effectiveness of the same six-week, low-cost exergame programme delivered via telerehabilitation (TR) and face-to-face (FF) in CP children. **Methods:** In this randomised controlled trial, 15 CP patients completed 18 sessions over 6 weeks. The TR group received remotely delivered sessions, whereas the FF control group completed in-person sessions with a physiotherapist. Outcomes were assessed at baseline; weeks 2, 4, and 6; and follow-ups at weeks 8 and 10. Postural control (centre-of-pressure sway area; CoP_sway_) was measured during eyes open (EO), eyes closed (EC), voluntary mediolateral sway to a 30 bpm auditory cue (EO/EC), and during exergames targeting mediolateral (ML-WS) and anteroposterior (AP-WS) weight-shifting. Timed Up and Go (TUG) and Modified Modified Ashworth Scale (MMAS) were also assessed. **Results**: At week 6, both TR and FF significantly reduced CoP_sway_ (TR: *p* = 0.001, EC; FF: *p* = 0.01, EO). TR also improved dynamic postural control (*p* < 0.05) and TUG scores (*p* = 0.03), with functional gains sustained until week 10. Between-group comparisons revealed that TR achieved significantly greater reductions in AP weight-shifting (SD_ML_, *p* = 0.001; V_ML_, *p* = 0.004) and TUG (*p* = 0.009) than FF, with these advantages persisting throughout follow-up as revealed by post hoc analysis. Conversely, only FF significantly reduced ankle muscle tone (MMAS, *p* = 0.05). TR demonstrated broader improvements in secondary CoP metrics and superior long-term retention of functional mobility gains. **Conclusions:** Both six-week exergame interventions led to improvements in postural control. This trial demonstrated that telerehabilitation is a viable, comparable alternative to face-to-face delivery. Long-term retention suggests both modalities are complementary, offering flexible solutions to enhance routine physiotherapy service pathways. These findings provide a basis for validating these models across larger clinical cohorts.

## 1. Introduction

Cerebral palsy (CP) is the most prevalent childhood-onset physical disability, with many children experiencing deficits in standing balance (postural control) [1,2]. Deficits in standing balance and sensory–motor integration can limit safe sitting, standing, transfers, and walking, restricting participation in daily activities and increasing caregiver burden [3,4]. Studies using posturographic outcome measures have long shown that children with CP exhibit altered postural sway, hence balance control, compared with typically developing peers, particularly when sensory conditions are challenged [1]. Importantly, deficits in postural control are linked to reduced functional performance among young people with CP, highlighting postural control as a meaningful focus for rehabilitation [5].

Despite this need, achieving sufficient dose and specificity of balance training in paediatric neurorehabilitation remains challenging. Therapy resources are limited, travel demands are high for families, particularly in rural areas, and conventional programmes can struggle to maintain adherence [6,7,8]. Exercise-based interventions can improve postural control in CP, yet evidence across approaches highlights variability in content, supervision, and dosage, hence there is a need for more standardised, scalable programmes with outcomes that reflect improvements in function [9,10,11]. These realities motivate interventional approaches that can deliver high-repetition practice, meaningful feedback, and sustained engagement while reducing the logistical barriers that often limit access to rehabilitation [12].

Exergames and virtual reality-based activities offer one such approach as they combine therapeutic movements with game-like goals, real-time feedback, and progression that can increase motivation and time-on-task in CP children [13,14]. Gamification of rehabilitation interventions is widely proposed as a tool to enhance engagement and learning by making practice more rewarding and repeatable [15]. For balance-focused rehabilitation, exergames can be designed to drive controlled weight-shifting and reactive balance responses during dynamic tasks within safe boundaries, potentially increasing the volume of task-specific practice beyond what is feasible in a typical physiotherapy session [12,13]. Since functional weight shifting underpins many daily activities and depends on effective sensorimotor integration (e.g., proprioceptive and somatosensory inputs) to maintain stability, targeting weight-shifting control is central to rehabilitation programmes aimed at translating gains to real-world participation.

In children with CP, the evidence supporting exergaming and interactive computer play is encouraging. Systematic reviews indicate that videogame-based interventions can improve balance and postural control, sometimes with medium to large effects [15,16]. Recent pooled evidence suggests that virtual reality exergames may benefit balance control and gross motor performance in young people with CP, reinforcing their promise as a rehabilitation strategy; it should be noted, however, that the effects on daily living ability were less consistent across studies [17]. Taken together, these findings suggest that exergames can be effective, but they also underline the need for well-controlled trials that specify dosage, delivery, and objective outcomes.

Telerehabilitation (TR) may further strengthen the clinical value of exergames by addressing the accessibility problem directly. TR enables home-based delivery, reduces travel burden, and may support continuity of care across disruptions to in-person services [18,19]. In paediatric neurorehabilitation, recent reviews suggest that tele-neurorehabilitation can improve motor outcomes, with particularly promising findings reported in children with CP and hemiplegia [20]. In CP specifically, randomised trials of structured TR home programmes have demonstrated improvements in outcomes related to activity and participation when added to usual care, supporting TR as a credible delivery model in paediatrics [21].

However, TR is not simply a change in setting since supervision, feedback, fidelity, and progression can all affect the effectiveness and efficiency of exergames [22,23,24]. Therefore, the key clinical question is not only whether exergames “work”, but whether they work similarly when delivered remotely compared with face-to-face (FF) implementation [24]. This gap has been explicitly recognised in the exergame TR literature [25]. Beyond paediatrics, it has been shown that in older adults, TR and FF delivery of a standardised exergame programme can both improve postural control, with modality-specific patterns across tasks, supporting the feasibility of meaningful head-to-head comparisons of delivery models [24,25].

Summarising, a rigorous comparison of TR and FF exergame delivery in children with CP has clear clinical and health-service relevance [12,14]. If TR produces comparable improvements to FF, it could expand access for families in remote or low-resource settings, support hybrid models that combine periodic in-person review with home-based training and improve continuity of care when clinical attendance is disrupted [18,19,26,27]. Further, trials that use objective posturographic measures (e.g., centre-of-pressure sway) during functional tasks (e.g., weight shifting) can standardise outcome assessment across sites and improve comparability between studies [1,11,12].

The trial examined whether the same six-week, low-cost exergame programme, delivered remotely through telerehabilitation (TR) or face-to-face (FF), produced comparable postural-control outcomes in children with spastic hemiplegic cerebral palsy. The guiding research question was: does TR delivery of this intervention produce postural-control outcomes similar to those observed with FF delivery in this population? Because both programmes delivered the same multisensory exergames, we hypothesised that TR and FF would produce similar improvements in postural control. Similar patterns of improvement would support TR as an accessible adjunct to physiotherapy for children with spastic hemiplegic CP. This could broaden rehabilitation delivery by offering a scalable remote pathway to address postural-control impairments when face-to-face care is limited or impractical.

## 2. Materials and Methods

### 2.1. Study Design and Sample Size

**Figure 1:**
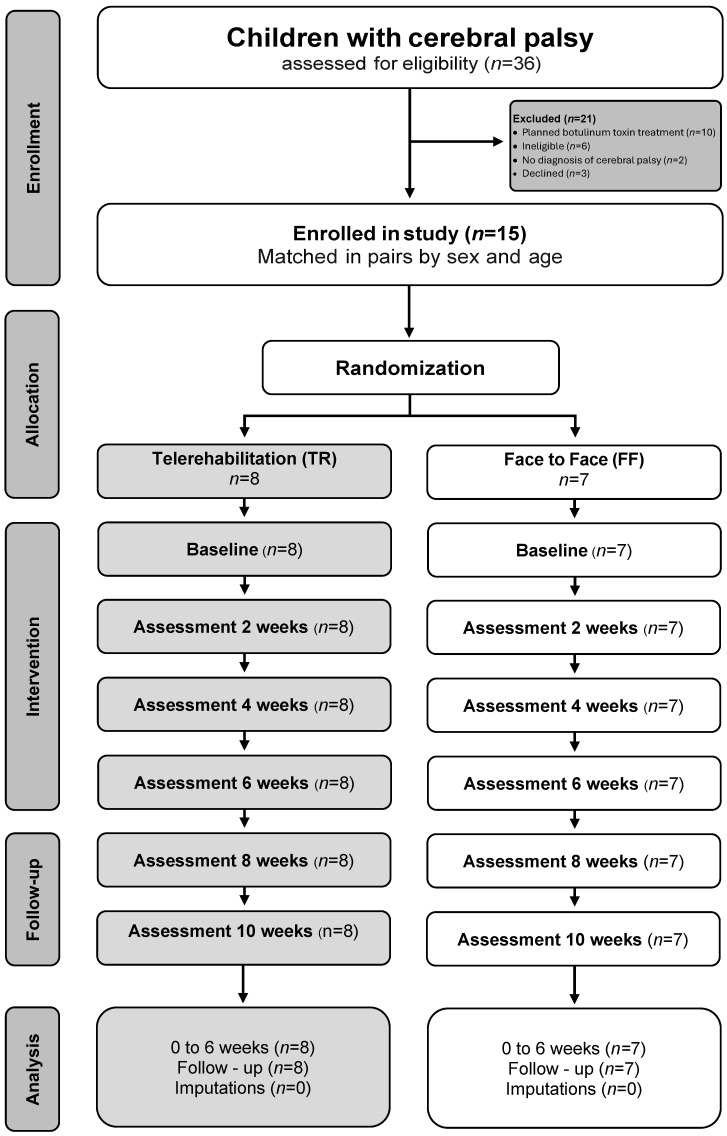
Participant flowchart for the prospective randomised controlled trial using matched-pair allocation in children with spastic hemiplegic cerebral palsy.

The study used a two-group randomised controlled design, with allocation concealed until participants were assigned to intervention arms [28]. Blinding participants to their assigned group was impractical because the two intervention delivery modes were inherently distinguishable [29]. Outcome assessors remained blinded to group allocation. In addition, the physiotherapist delivering the FF sessions was given only the information required to administer the exergame programme and was not informed about the TR arm; the two delivery formats were also implemented at different sites to minimise cross-condition awareness. Blinding was further supported by conducting TR and FF sessions in separate locations. Two independent assessors, both clinicians external to the research team, conducted the outcome assessments. Prospective trial registration and publication of the study protocol were undertaken to support transparency in the planned data collection and analytical methods [12] (Figure 1). To enhance transparency and reproducibility, the interventions were characterised using the TIDieR framework. Furthermore, this trial was reported in compliance with CONSORT standards [28].

The initial sample-size calculation was informed by clinical data and previously reported measures of postural balance [22,23]. However, COVID-19-related disruption to data collection curtailed recruitment, and the prespecified sample size could not be achieved. Nevertheless, this did not alter the randomised controlled design of the study. Given the final feasible sample, we estimated that a mean difference of 31.7 cm^2^ in the primary outcome of centre-of-pressure (CoP) sway area, assuming a standard deviation of 20.68 cm^2^, is necessary to detect a substantial clinically relevant effect. Using a two-sided alpha of 0.05, 80% power, and allowing for 6% attrition, the minimum sample was estimated at 15 participants (8 in one group and 7 in the other). These calculations were performed using GRANMO v.7.12 (Institut Municipal d’Investigació Mèdica, Barcelona, Spain).

### 2.2. Recruitment

Children and adolescents with congenital spastic hemiplegic cerebral palsy were recruited from the Rehabilitation Institute in central Chile, an outpatient service providing rehabilitation for people with neurological conditions, including children with CP. Participant screening and enrolment were conducted by two physiotherapists and one medical doctor, who continued recruitment until the target sample size was achieved. Eligible participants were boys or girls aged 7–14 years with spastic hemiplegic CP, classified as level I or II on the Gross Motor Function Classification System—Expanded and Revised (GMFCS-ER), and with mild or no cognitive impairment. Children were excluded if they had vestibular dysfunction, uncorrected visual impairment, severe epilepsy, or previous experience using the Nintendo Wii.

The study was conducted in accordance with the ethical standards set out in the Declaration of Helsinki, the Belmont Report, and the CIOMS Guidelines, as well as applicable Chilean legislation. Approval was granted by the Ethics Committee of the University of Talca (Ref. No. 24-2018). The trial was also registered with the Australian and New Zealand Clinical Trials Registry, which is included in the International Clinical Trials Registry Platform (ICTRP), under registration number ACTRN12621000117819. All study procedures complied with the relevant ethical guidelines and regulatory requirements. Parents or legal guardians provided written informed consent for each participant. In addition, written assent was obtained from children aged 7 years and older, whereas younger children provided verbal assent.

### 2.3. Randomization

Participants were assigned in equal proportions to the TR or FF group using a computerised web-based randomisation system. After allocation, each participant was told their assigned group and received a detailed explanation of the intervention procedures. Periodic consultations were conducted to clarify aspects of the intervention and support adherence. Outcome assessors were kept blinded to group allocation for the duration of the study. To maintain blinding during analysis, an independent research assistant entered the data into confidential datasheets. The participant flow is presented in Figure 1.

### 2.4. Posturographic and Clinical Measurements

Postural and clinical outcomes were assessed at baseline; weeks 2 and 4 during the intervention; week 6 at intervention completion; and weeks 8 and 10 as follow-up assessments. Each postural task and clinical test was repeated three times, with the mean value used for analysis to improve measurement reliability. At each assessment point, the full set of postural and clinical evaluations required approximately 20–30 min per participant. Measurements for each group were carried out independently at the Human Motor Control Laboratory (Universidad de Talca, Chile) by blinded assessors to ensure the objectivity of the results.

#### 2.4.1. Postural Tasks

**Figure 2:**
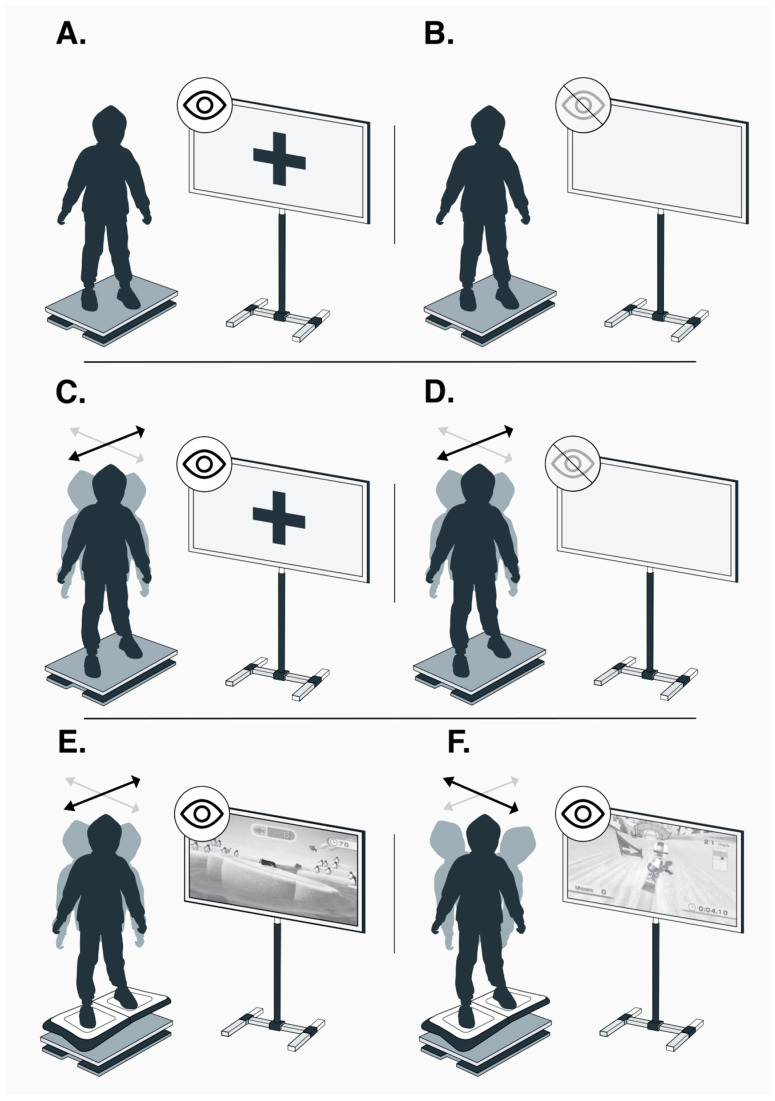
Six postural tasks: two static tasks, performed with (**A**) eyes open (EO) and (**B**) eyes closed (EC); and four dynamic tasks, comprising voluntary mediolateral sway paced by a metronome at 30 bpm with (**C**) eyes open (ML-EO30) and (**D**) eyes closed (ML-EC30), (**E**) a mediolateral weight-shifting exergame (ML-WS), and (**F**) an anterior–posterior weight-shifting exergame (AP-WS). Grey and black arrows indicate mediolateral and anterior–posterior displacement, respectively. Image credit: Valeska Gatica-Rojas, PhD.

CoP displacement was collected using an AMTI OR6-7 force plate (Watertown, MA, USA) sampled at 200 Hz. Recordings were obtained during static standing with (i) eyes open (EO) and (ii) eyes closed (EC). For these trials, participants stood on the force plate with feet shoulder-width apart and arms resting by their sides. CoP displacement was also measured during voluntary mediolateral sway paced by a metronome at 30 bpm under (iii) EO (ML-EO30) and (iv) EC (ML-EC30) conditions; during (v) mediolateral weight shifting (ML-WS) while playing Penguin; and during (vi) anterior–posterior weight shifting (AP-WS) while playing Snowboard. For the ML-WS and AP-WS tasks, the Nintendo Balance Board was positioned on top of the force plate. The EO and EC conditions represented standard static postural-control assessments, while the remaining four tasks were dynamic assessments designed to examine control in the mediolateral and anterior–posterior planes. Each assessment was completed three times, with trials lasting 30 s. The posturography setup for the EO, EC, and dynamic conditions is shown in Figure 2. Across all trials, CoP signals were low-pass filtered at 40 Hz using a second-order Butterworth filter, and CoP outcomes were then calculated in Python 3.11.13.

#### 2.4.2. Clinical Measures

Modified Modified Ashworth Scale (MMAS) for lower limbs was used to assess whether changes in muscle tone in ankle plantar flexors musculature, respectively [30].

The Timed Up and Go (TUG) records, in seconds, how long a child takes to stand from a chair, walk 3 metres, turn around at a marked point, walk back, and sit down again. In CP children, the TUG is an excellent indicator of transitional ability and gait stability [31].

### 2.5. Intervention Modalities

Participants in both groups undertook an identical exergame-based therapy programme using a Nintendo Wii and Wii Balance Board. The intervention involved 18 sessions over six weeks, delivered three times per week, with each session lasting 25 min.

The programme comprised three exercise sets designed to challenge balance control in multiple planes of movement. The first two sets included Snowboard, Penguin Slide, and Super Hula Hoop, whereas the third set used the Yoga game. Participants rested in a sitting position for approximately 2 min between sets. During the first set, participants performed the games while standing comfortably with their arms by their sides; during the second set, the same games were repeated with hands placed on the waist. The final set required participants to maintain a relaxed standing position during the Yoga game, initially with eyes open and then with eyes closed. Examples of the exergames are shown in Figure 2E,F.

To encourage adherence, participants in both the TR and FF groups were contacted by telephone to remind them of scheduled sessions. The intervention protocols remained unchanged for the duration of the study.

#### 2.5.1. Telerehabilitation (TR)

**Figure 3:**
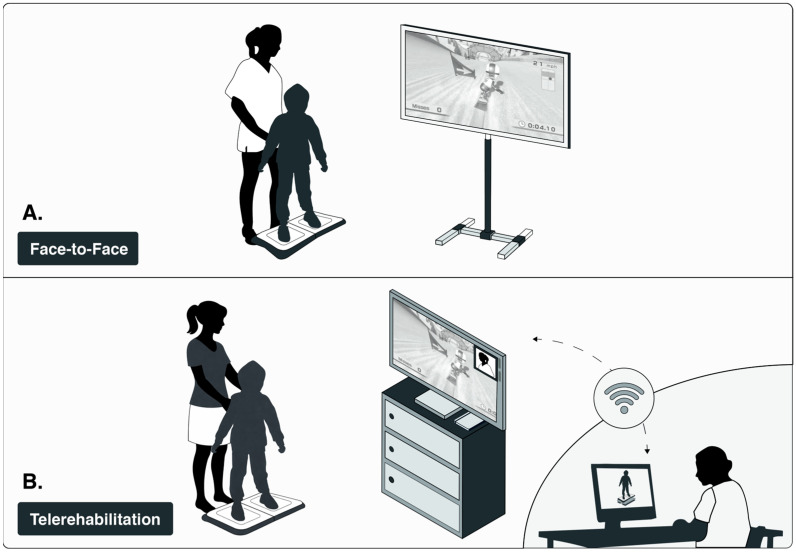
Intervention Modalities: (**A**) face to face and (**B**) telerehabilitation. Image credit: Valeska Gatica-Rojas, PhD.

In the TR, the intervention was delivered by the participants’ parents or caregivers in their homes; these individuals had received prior training in the correct and safe delivery and assistance of the exergame activities. Parents or caregivers served as peer monitors to support session delivery and technology use. During the first three weeks, they provided tactile and verbal cues to help participants perform the exergames correctly. During the final three weeks, support was restricted to verbal cueing. When further assistance was required, the physiotherapist provided parents or caregivers with real-time remote instructions using verbal and/or visual guidance (Figure 3). The project team managed connectivity and other technical support needs.

#### 2.5.2. Face-to-Face (FF)

The FF exergame sessions took place at the Human Motor Control Laboratory of the Universidad de Talca, Chile.

During the first three weeks, the physiotherapist provided both tactile and verbal cues to guarantee that the exergames were performed with correct technique and optimal safety. Over the final three weeks, this support was scaled back to verbal guidance only; this approach aimed to foster greater participant autonomy while ensuring that the quality of exercise remained consistent (Figure 3).

### 2.6. Outcome Measurement

The primary outcome was CoP sway area (CoP_sway_), a commonly used and reliable measure of balance impairment during postural tasks [30,31]. CoP_sway_ represents the overall CoP trajectory across the mediolateral (ML) and anterior–posterior (AP) directions. It provides an integrated measure of the capacity of the postural control system to maintain upright stability, with larger values reflecting poorer balance control.

Secondary outcomes comprised the standard deviation of CoP displacement in the mediolateral and anterior–posterior directions (SD_ML_ and SD_AP_), representing the variability of CoP movement, and CoP velocity in the same directions (V_ML_ and V_AP_), reflecting the rate of postural adjustments. Higher values for these measures indicate poorer balance control. In particular, delayed or less effective postural responses may result in larger CoP excursions, followed by faster compensatory movements to maintain stability [30]. In alignment with the prespecified protocol [12], diverse secondary outcomes were analysed to capture clinical effects and complement the primary findings [32].

The MMAS and TUG completion time were also evaluated as clinical secondary outcomes.

### 2.7. Statistical Analysis

Descriptive summaries were generated for all demographic and clinical characteristics. Baseline differences between groups were examined using unpaired *t*-tests for continuous variables and Fisher’s exact test for categorical variables. The Shapiro–Wilk test was used to evaluate normality, while Levene’s test assessed equality of variances. Because the sample was small and several outcomes were not normally distributed, the primary statistical analyses were conducted using non-parametric approaches. Within-group changes across assessment time points were analysed using Friedman’s ANOVA. When indicated, Wilcoxon signed-rank tests were applied for post hoc pairwise comparisons. Between-group comparisons at specific time points, including the post-intervention assessment at week 6, were conducted using the Mann–Whitney U test. To evaluate the magnitude of change in CoP parameters from baseline (week 0) to post-intervention (week 6), and subsequently to the week 10 follow-up, Kendall’s W was calculated. While the overall level of statistical significance was set at *p* ≤ 0.05, a Bonferroni adjustment was applied to the two prespecified contrasts addressing immediate intervention and retention effects; for these analyses, the significance threshold was set at *p* < 0.025 (0.05/2) to maintain statistical stringency. All statistical analyses were performed using a custom script written in Python 3.11.13, which is publicly accessible on GitHub (https://github.com/JS-75/AlgosCH; accessed 20 January 2026).

## 3. Results

### 3.1. Participants

Nineteen children with spastic hemiplegic CP were identified as potentially eligible. Of these, 15 were enrolled and randomised to the TR group (n = 8) or the FF group (n = 7). The two groups were comparable across the demographic variables assessed in the study (Table 1) and showed similar baseline values for all outcome measures.

Compliance was monitored through attendance logs and exergame records, maintained by the peer monitor for the TR group and the therapist for the FF group. Overall, compliance was 100%. No participants were lost to follow-up, resulting in no missing data; consequently, the intention-to-treat analysis was performed on the full randomised cohort. Given the zero-attrition rate, the achieved sample size yielded a post hoc statistical power of 84.2% at a significance level of alpha = 0.05.

### 3.2. Primary Outcome

#### 3.2.1. Between-Group Differences at 6-Week Time Point (End of Intervention)

At the end of the 6-week intervention, CoP_sway_, measured across six postural tasks (EO, EC, ML-EO30, ML-EC30, ML-WS, and AP-WS), showed no significant differences (*p* > 0.05) between the TR and FF groups. These findings suggest that both exergame programmes were associated with a broadly similar pattern of effects, with no clear advantage of one delivery mode over the other. Furthermore, no significant between-group differences (*p* > 0.05) were observed during the follow-up assessments at weeks 8 and 10.

#### 3.2.2. Effects of TR and FF over Time

Regarding within-group effects over time, the TR group showed significant reductions in CoP_sway_ at week 6 compared with baseline under the EC condition (F = 17.71, *p* = 0.001; strong, W = 0.565), the EO condition (F = 14.23, *p* = 0.012; moderate, W = 0.486), the ML-EO30 task (F = 14.13, *p* = 0.03; weak, W = 0.286), and the AP weight-shifting task (Snowboard; F = 14.01, *p* = 0.04; moderate, W = 0.371). After applying the Bonferroni-adjusted threshold for the two prespecified contrasts (*p* < 0.025), the EC and EO findings remained significant, whereas the ML-EO30 and AP weight-shifting findings did not. In addition, the reduction in CoP_sway_ under the EC condition was maintained at the week 8 follow-up (F = 14.45, *p* = 0.016). Across the remaining dynamic tasks, effect sizes in the TR group ranged from moderate in ML-EC30 (W = 0.323) and ML weight shifting (W = 0.307) to weak in ML-EO30 (Table 2).

In the FF group, CoP_sway_ decreased significantly at week 6 compared with baseline during EO (F = 16.24, *p* = 0.01; moderate, W = 0.480) and EC (F = 14.51, *p* = 0.021; strong, W = 0.537). Both findings remained significant under the Bonferroni-adjusted threshold. Although no significant post hoc baseline-to-week-6 effects were observed for the dynamic postural tasks, effect sizes suggested heterogeneous time-related patterns, ranging from strong in ML-EO30 (W = 0.501) and moderate in ML-EC30 (W = 0.382) to weak in AP weight shifting (W = 0.125) and very weak in ML weight shifting (W = 0.060).

### 3.3. Secondary Outcomes

#### 3.3.1. Between-Group Differences at 6-Week Time Point (End of Intervention)

A summary with main findings for the secondary outcome measure between groups and for each group over time is presented in Table 3 whereas full statistical results are provided in the Supplementary Table S1. Secondary outcomes were broadly consistent with the primary outcome, with no significant between-group differences at week 6 in most postural tasks. The only exception was the dynamic AP-WS, in which the TR group showed greater reductions than the FF group in SD*_ML_* (*p* = 0.001) and V*_ML_* (*p* = 0.004). These differences were partly retained at follow-up, remaining significant for SD*_ML_* at week 8 (*p* = 0.03) and for V*_ML_* at week 10 (*p* = 0.04). The TR group also showed lower TUG scores than the FF group at week 6 (*p* = 0.009), with this difference maintained at weeks 8 and 10 (both *p* = 0.01). As for the secondary outcome measure MMAS, no between-group differences were found.

#### 3.3.2. Effects of TR and FF over Time

At week 6, the TR group showed significant improvements from baseline in several postural secondary outcomes. In EO, SD_ML_ decreased (F = 13.96; *p* = 0.038), with a strong overall time effect (W = 0.519), and V_AP_ decreased (F = 14.03; *p* = 0.025), with a moderate overall time effect (W = 0.462); these effects were retained to week 8 for SD_ML_ (*p* = 0.025) and to week 10 for V_AP_ (*p* = 0.04). In EC, SD_ML_ (F = 17.10; *p* = 0.001), V_ML_ (F = 13.88; *p* = 0.04), and V_AP_ (F = 15.54; *p* = 0.003) decreased, all showing strong overall time effects (W = 0.605, 0.565, and 0.582, respectively); follow-up effects were retained for SD_ML_ at week 8 (*p* = 0.004) and for V_AP_ at weeks 8 and 10 (*p* = 0.03). In ML-EO30, SD_AP_ (F = 15.06; *p* = 0.001) and V_AP_ (F = 14.02; *p* = 0.03) decreased, with strong (W = 0.519) and moderate (W = 0.339) overall time effects, respectively, although only SD_AP_ remained improved at week 10. In ML-EC30, SD_ML_ (F = 13.92; *p* = 0.04), SD_AP_ (F = 15.23; *p* = 0.002), V_ML_ (F = 14.42; *p* = 0.016), and V_AP_ (F = 14.45; *p* = 0.016) decreased, with weak (W = 0.275), strong (W = 0.605), moderate (W = 0.348), and moderate (W = 0.426) overall time effects, respectively; only SD_AP_ and V_AP_ showed retained effects. In the dynamic AP weight-shift task, SD_ML_ (*p* = 0.016) and V_ML_ (*p* = 0.0108) decreased at week 6, both with moderate overall time effects (W = 0.347 and 0.388, respectively), but these effects were not maintained. For clinical secondary outcomes, the TR group improved on the TUG at week 6 relative to baseline (F = 13.02; *p* = 0.03), with gains retained at weeks 8 (*p* = 0.011) and 10 (*p* = 0.025), whereas MMAS did not change.

The FF group showed fewer and less sustained changes. In EO, SD_ML_ (F = 14.41; *p* = 0.013) and SD_AP_ (F = 15.16; *p* = 0.004) decreased at week 6, both with strong overall time effects (W = 0.525 and 0.509, respectively), although the SD_AP_ effect was not maintained. In ML-EC30, SD_ML_ (*p* = 0.01) and V_ML_ (*p* = 0.02) decreased, with strong (W = 0.593) and moderate (W = 0.450) overall time effects, respectively, with retention only for SDML at week 8 (*p* = 0.013). During the dynamic ML weight-shift task, SD_ML_ increased at week 6 relative to baseline (*p* = 0.032), despite only a weak overall time effect (W = 0.291). In contrast, the FF group showed reduced ankle muscle tone on the MMAS at week 6 (F = 11.56; *p* = 0.05), with this effect retained at week 10 (*p* = 0.05), but no significant TUG change (Supplementary Table S2).

### 3.4. Adverse Events

No adverse events were recorded in either group at any time point.

## 4. Discussion

This randomised controlled trial compared telerehabilitation (TR) and face-to-face (FF) delivery of the same six-week, low-cost exergame programme for improving postural control in children with spastic hemiplegic CP. To the best of our knowledge, this is the first study to directly compare these delivery modalities, addressing service priorities of increasing access to high-dose training, reducing travel burden, and supporting continuity of care using engaging, low-cost exergame technology. Furthermore, the inclusion of longitudinal follow-up enabled evaluation of treatment retention, providing clearer insight into the longer-term sustainability of effects for each delivery modality.

Across analyses, our findings indicate that a six-week exergame intervention, delivered via either telerehabilitation (TR) or face-to-face (FF) therapy, was associated with significant improvements in postural control. Both groups demonstrated gains across primary and secondary outcomes in postural tasks and clinical assessments. To ensure the reliability of these results, the analysis was conducted within a rigorous framework that accounts for the multiplicity of outcomes and repeated assessments. This was achieved by anchoring the interpretation to prespecified, clinically relevant contrasts—specifically baseline to post-intervention and post-intervention to follow-up—rather than an exhaustive array of pairwise comparisons. Such a targeted approach reinforces the validity of the observed effects, ensuring that the primary temporal patterns reflect genuine clinical shifts rather than statistical artefacts. Consequently, the findings provide a basis for observing that the TR group showed superior retention of gains, particularly in static and more demanding dynamic postural tasks.

Our analysis of the trial’s primary outcome (CoP_sway_) across the six-week intervention revealed that each delivery modality elicited task-specific effects. Whereas FF significantly improved postural control during eyes-open (EO) standing, TR was similarly effective for stability under eyes-closed (EC) conditions. These patterns suggest that TR and FF may be complementary delivery options and that contextual factors surrounding training at home and in the clinic may differentially influence how visual information is used for balance control during postural tasks. Further, whereas the TR improved EO, FF improved EC postural control from week 2 to the end of the intervention, with gains maintained to week 8 (2 weeks post-intervention). Notably, only TR demonstrated greater improvements in CoP_sway_ during more demanding dynamic tasks, specifically ML-EO30 and the dynamic AP weight-shifting exergame. These findings may indicate more efficient balance control, with motor strategies that minimise displacement in directions that are not demanded by the task [24,33].

Before interpreting the secondary outcomes, it is important to note that these analyses are best viewed as supportive rather than confirmatory and should therefore be considered with appropriate caution. TR produced broader and more sustained secondary improvements than FF, suggesting a wider effect on balance control. Under eyes-open conditions, both groups improved at the end of the intervention, but only TR retained these gains over time [34,35]. More notably, only TR improved postural control under eyes-closed conditions, which may reflect better sensory reweighting toward proprioceptive and vestibular inputs when visual information is unavailable [36]. This is noteworthy because the exergames relied heavily on visual feedback, suggesting that proprioceptive aspects of balance can still be trained in visually enriched tasks, consistent with cross-sensory effects reported in other populations [24,37]. TR also showed sustained improvements during mediolateral tasks, which may indicate optimisation of hip and trunk-pelvis strategies that are more dependent on proximal control than the ankle-dominant strategies typically used for anteroposterior sway [38]. Although voluntary AP weight shifting with auditory cueing was not assessed, improvements during AP weight-shift tasks may also reflect better control of the musculature involved in these movements [24,38].

By contrast, FF showed fewer and generally less sustained secondary improvements. One explanation is that closer physiotherapist supervision constrained compensatory movements, effectively reducing sway in directions not directly required by the task. The only result that deviated from this pattern was increased sway during one mediolateral weight-shift task; although the exact mechanism remains to be fully elucidated, this likely reflects a trade-off between movement strategies or adaptive exploration of motor solutions rather than a deficit in balance control [39]. TR also led to sustained improvements in TUG, suggesting retention of functional mobility benefits. Training in the home environment facilitated the transfer of gains to everyday mobility by aligning practice with real-life contexts and mitigating logistical barriers [40,41], while family encouragement further bolstered engagement [42]. In contrast, the reduction in ankle muscle tone observed exclusively in FF underscores the added value of in-person clinician input, including real-time feedback and physical guidance; this specialised interaction fosters more accurate weight shifting and enhances somatosensory and proprioceptive input [43,44,45,46]. Overall, these findings reveal modality-specific advantages, with TR driving motivational and contextual benefits, while FF delivers superior clinical precision in movement. Future studies should determine whether these improvements translate to enhanced daily life participation in CP children [47,48].

Compared with previous RCTs in children with CP, our findings should be contextualised within a key methodological distinction. While most earlier trials contrasted exergame or VR-based interventions with conventional therapy or another non-equivalent control, our study compared the same exergame programme across two distinct modalities: telerehabilitation and face-to-face care. In this framework, the absence of significant between-group differences in CoP sway area aligns with established literature. Rather than indicating a lack of effect, this suggests that when exergame content, frequency, and duration are held constant, both delivery modes effectively support improvements in postural control. This interpretation reinforces previous evidence showing that exergame interventions enhance posturographic balance outcomes in children with CP, even when clear between-group superiority is not uniformly reported across the literature, particularly in smaller-scale studies [49,50,51].

Our study aligns with several methodological features of previous RCTs, including the use of focussed clinical cohorts, game-based balance interventions, and posturographic outcomes. Most established trials have utilised targeted sample sizes, typically ranging from 15 to 32 participants [22,49,50,51], reflecting the specialised nature of this clinical population. Likewise, various interventions employed Nintendo Wii or similar balance-oriented platforms, while others utilised active videogames or custom gaming systems rather than conventional therapy alone [22,49,50,52]. The use of posturographic measures based on force platforms or centre-of-pressure variables was consistent across these studies, ensuring they are more methodologically comparable to our trial than studies relying only on clinical balance scales. Consequently, our findings extend prior research by suggesting that exergame-related postural benefits may be retained even when the key comparison is not exergames versus conventional therapy, but delivery modality of the same exergame dose.

This RCT contributes to the growing evidence that exergame-based interventions enhance balance control in children with CP and suggests that telerehabilitation (TR) may be a feasible mode of delivery. We recognise that TR and face-to-face (FF) therapy involve distinct operational contexts, particularly regarding supervision and caregiver involvement. To harmonise these modalities, caregivers received training before the intervention and remote physiotherapist support was available on request throughout the programme. While the study focused on real-world application, the inherent variability in exercise execution and environmental fidelity within the TR arm provides an authentic representation of home-based rehabilitation. Although this introduces a degree of contextual diversity, such factors are intrinsic to the practical implementation of TR and reflect the ecological setting for which these interventions are designed. Therefore, the observed outcomes offer a pragmatic assessment of how these delivery modes perform in routine clinical and domestic practice.

Our primary motivation was to enhance access to rehabilitation while alleviating the economic and logistical burden of travel for families. We recognise, however, that contextual factors—such as the urban-versus-rural distribution between groups—contribute to the complexity of the intervention’s landscape. Rather than viewing this as a confounding variable, it reflects the real-world environment where telerehabilitation (TR) is most impactful. Families in rural and underserved areas are precisely those who stand to gain the most from approaches that bridge geographical gaps [27]. This underscores the necessity of evaluating interventions within the pragmatic realities of home-based care. Thus, TR emerges as a vital and accessible pathway for ensuring rehabilitation continuity, particularly when conventional in-person services are geographically or logistically constrained.

### Strengths and Limitations of the Study

Strengths of the present study include the novel head-to-head comparison of two scalable delivery models within a blinded, randomised controlled design. Internal validity and reproducibility were strengthened by combining validated clinical outcomes with objective posturographic measures. Postural control was assessed across a broad task spectrum, from static standing to more demanding dynamic challenges. Reductions in CoP_sway_ were interpreted as improved stability and more task-optimised balance control, supported by consistent patterns across both primary and secondary CoP metrics and clinical measures.

A notable methodological strength was the standardised progression of clinical guidance across both delivery modes. By providing combined tactile and verbal guidance during the initial three weeks and transitioning to exclusively verbal cues for the final three weeks in both groups, the study ensured a controlled withdrawal of physical assistance. The absence of significant between-group differences suggests this structured approach was equally effective in both contexts, demonstrating that the transition to verbal-only guidance did not preclude significant postural improvements, whether delivered in-person or remotely.

Several considerations regarding the study’s scope should be noted. Although the sample size was determined a priori for the primary outcome, recruitment was impacted by the unprecedented constraints of the COVID-19 pandemic. While this resulted in a more focussed cohort, the 6-week intervention yielded detectable and significant effects within this group, providing an indication of the programme’s effectiveness. Future research with expanded, multi-centre cohorts could further refine the precision of between-group estimates, particularly for secondary outcomes. Furthermore, as recruitment was concentrated in a specific geographical region in Chile, these findings offer insights that are valuable for the local implementation of telerehabilitation, serving as a foundation for larger-scale validation across diverse clinical settings.

Additionally, while the Nintendo Wii is a low-cost platform, its technical capabilities are surpassed by newer systems. This highlights ongoing challenges in developing affordable, clinically effective exergaming solutions, an area our group is actively investigating [53]. Finally, our cohort included children with spastic hemiplegic CP with mild or no cognitive impairment. While this likely supported adherence and reduced confounding related to task comprehension, it may limit applicability to children with greater cognitive deficits. Future studies should extend this work to broader CP phenotypes, including non-spastic subtypes and children with more severe motor and cognitive involvement. 

## 5. Conclusions

In this trial, both telerehabilitation (TR) and face-to-face (FF) delivery of a low-cost exergame programme were associated with improvements in postural control in children with spastic hemiplegic CP, with no clear between-group advantage observed. Some gains were retained beyond the intervention period, suggesting that TR may be a useful adjunct to existing physiotherapy pathways. In this context, TR and FF may be better understood as complementary rather than competing approaches, with possible value within hybrid models of care tailored to family needs and service capacity.

## Figures and Tables

**Table 1:** Baseline characteristic of the participants.

Demographics		TR (n = 8)	FF (n = 7)	*p*-Value
Age (years), mean (SD)		9.8 (2.3)	10.1 (2.8)	0.825 ^a^
Sex, *n* (%)	Female Male	5 (62.5) 3 (37.5)	4 (57.14) 3 (42.9)	1.000 ^b^
Weight (kg), mean (SD)		40.68 (9.46)	39.64 (12.03)	0.857 ^a^
Height (m), mean (SD)		1.38 (0.16)	1.38 (0.18)	1.000 ^a^
BMI (kg/m^2^), mean (SD)		21.36 (4.04)	20.81 (3.86)	0.792 ^a^
Hemiplegia *n* (%)	Left side	4 (50.00)	2 (28.57)	0.608 ^b^
	Right side	4 (50.00)	5 (71.43)	
GMFCS *n* (%)	Level 1	3 (37.50)	3 (42.85)	1.000 ^b^
	Level 2	5 (62.50)	4 (57.14)	
GMFCS-ER *n* (%)	Level 1	2 (25.00)	3 (42.86)	0.609 ^b^
	Level 2	6 (75.00)	4 (57.14)	
Other diagnoses *n* (%)	Corrected vision	6 (75.00)	5 (71.43)	1.000 ^b^
	Corrected hearing	1 (12.50)	1 (14.29)	
* Prior surgery *n* (%)	Right lower limb	2 (25.00)	1 (28.57)	1.000 ^b^
	Left lower limb	1 (12.50)	1 (14.28)	
Geographic location *n* (%)	Mayor city	8 (100)	0	<0.001 ^b^
	Rural town	0	7 (100)	
Socioeconomic level *n* (%)	Middle	6 (75.00)	5 (71. 43)	1.000 ^b^
	Low	2 (25.00)	2 (28.57)	

^a^ Unpaired *t*-tests. ^b^ Fisher’s exact test. BMI: Body Mass Index; GMFCS: Gross Motor Function Classification System; GMFCS-ER: Expanded and Revised Gross Motor Function Classification System. FF: face to face group; TR: telerehabilitation group. * Prior surgeries were gastrocnemius tenotomies at least 2 years prior to the study.

**Table 2:** Primary outcome measure during the six postural tasks for both groups over time.

			Baseline	Mid-Intervention		End Intervention	Follow-Ups		
			Week 0	Week 2	Week 4	Week 6	Week 8	Week 10	*p*-Value ^a^ (Kendall’s W)
	Task	Group	Median [IQR]	Median [IQR]	Median [IQR]	Median [IQR]	Median [IQR]	Median [IQR]	
**CoP_sway_ (cm^2^)**	**EO**	**FF**	436.90	631.20	348.90	119.10	411.20	196.60	<0.01 (0.48)
			[332.40–651.20]	[388.10–826.60]	[251.80–562.40]	[86.70–122.80]	[281.80–579.50]	[129.20–610.90]	
		**TR**	795.20	812.10	470.60	153.20	142.60	216.60	<0.01 (0.49)
			[466.40–1187.40]	[259.60–1440.00]	[364.80–581.10]	[109.70–196.20]	[85.60–421.00]	[174.40–400.40]	
	**EC**	**FF**	514.80	967.20	679.10	177.30	211.60	375.90	<0.01 (0.54)
			[339.00–665.30]	[519.20–1402.00]	[304.20–1673.10]	[85.40–442.00]	[129.30–494.20]	[171.30–711.80]	
		**TR**	726.60	529.40	469.30	260.30	194.70	357.10	<0.01 (0.57)
			[499.70–1334.70]	[470.10–795.80]	[299.40–617.70]	[171.10–326.30]	[142.10–502.10]	[158.70–491.40]	
	**ML-EO30**	**FF**	7476.00	6729.80	9441.60	4459.80	5060.20	5458.40	<0.01 (0.50)
			[6182.40–10,655.30]	[5906.50–9870.80]	[7411.30–12,227.20]	[3830.20–5339.10]	[2790.10–6478.40]	[3922.60–5972.60]	
		**TR**	10,707.10	8794.50	10,033.60	5713.10	5873.60	8448.60	0.04 (0.29)
			[10,156.10–12,981.20]	[7296.40–9978.60]	[5390.30–12,298.00]	[4354.00–8237.60]	[4566.00–7398.80]	[5336.40–10,557.60]	
	**ML-EC30**	**FF**	8249.50	8585.10	8091.80	4802.80	6297.30	5156.50	0.02 (0.38)
			[7784.70–8917.60]	[5565.30–9816.90]	[5800.80–13,072.10]	[3664.90–5773.70]	[5138.20–6561.10]	[3729.70–7047.70]	
		**TR**	10,318.30	9762.60	8238.40	5804.80	7064.20	6475.20	0.02 (0.32)
			[8596.00–13,291.30]	[6350.00–11,104.90]	[6049.20–11,572.90]	[4047.40–8460.80]	[5714.40–9421.30]	[5696.60–10,791.00]	
	**ML-WS**	**FF**	13,712.10	10,745.00	12,649.50	11,551.90	11,635.60	12,635.70	0.84 (0.06)
			[8362.50–15,272.50]	[9153.80–16,667.40]	[12,051.20–14,924.50]	[9235.80–14,386.70]	[10,562.90–13,525.80]	[9571.90–14,307.10]	
		**TR**	13,216.10	12,819.50	15,694.60	11,284.00	11,792.20	12,668.50	0.03 (0.31)
			[10,263.80–18,989.90]	[9873.60–15,605.20]	[11,757.50–17,558.50]	[6533.70–13,274.40]	[9690.70–13,983.60]	[11,850.10–13,845.00]	
	**AP-WS**	**FF**	10,373.40	8884.10	14,923.90	7217.20	7337.00	9932.80	0.50 (0.13)
			[8478.80–12,048.50]	[7917.20–12,008.70]	[9141.40–16,468.80]	[5637.80–13,123.20]	[6455.80–11,479.20]	[8573.00–11,994.20]	
		**TR**	14,278.50	9247.00	10,317.40	5792.70	6764.70	6582.50	0.01 (0.37)
			[7842.60–16,247.70]	[5957.00–14,269.80]	[8650.10–14,472.80]	[4460.90–8517.40]	[5725.50–7837.70]	[5338.70–7581.70]	

^a^ Friedman’s one-way ANOVA. W: Kendall’s W effect size; IQR: interquartile range; FF: face to face; TR: telerehabilitation; EO: eyes open; EC: eyes closed; ML-EO30: Mediolateral weight shifting with EO at 30 bpm; ML-EC30: Mediolateral weight shifting with EC at 30 bpm; ML-WS: mediolateral weight-shifting while playing Penguin; AP-WS: anteroposterior weight-shifting while playing Snowboard.

**Table 3:** Summary of main findings for the secondary outcome measure between groups and for each group over time.

	EO	EC	ML-EO30	ML-EC30	ML-WS	AP-WS	TUG	MMAS-R	MMAS-L
**FF**	✓✶	✓✶	✓✶	✓✶	-	✓	✓✶	✓✶	-
**TR**	-	-	-	✓✶	✓	-	-	✓✶	-
**Group effect**	-	-	-	-	-	♦	♦	-	-

FF: face to face; TR: telerehabilitation; EO: eyes open; EC: eyes closed; ML-EO30: Mediolateral weight shifting with EO at 30 bpm; ML-EC30: Mediolateral weight shifting with EC at 30 bpm; ML-WS: mediolateral weight-shifting while playing Penguin; AP-WS: anteroposterior weight-shifting while playing Snowboard; (✓) indicates significant differences between baseline and week 6 (end of intervention), (✶) indicate significant differences between baseline and follow-ups, (♦) indicates significant between groups differences at week 6 (end of trial). MMAS: Modified Modified Ashworth Scale; TUG: Timed Up and Go; R: right; L: left.

## Data Availability

Data will be available on request to the corresponding author due to privacy reasons.

## Supplementary Materials

The following supporting information can be downloaded at: https://www.mdpi.com/article/10.3390/medsci14020246/s1, Table S1: Secondary outcome measures during six postural tasks for both groups over time; Table S2: Secondary clinical outcome measures during two clinical tests for both groups over time.

## Abbreviations

The following abbreviations are used in this manuscript:

TR	Telerehabilitation
FF	Face-to-face
CoP	Centre-of-pressure
EO	Eyes Open
EC	Eyes Closed
ML-EO30	Mediolateral weight shifting with EO at 30 bpm
ML-EC30	Mediolateral weight shifting with EC at 30 bpm
ML-WS	Mediolateral weight-shifting while playing Penguin
AP-WS	Anteroposterior weight-shifting while playing Snowboard
MMAS	Modified Modified Ashworth Scale
TUG	Timed Up and Go
CoP_sway_	CoP sway area
SD*_ML_*	Standard deviation of the CoP medial–lateral
SD*_AP_*	Standard deviation of the CoP anterior–posterior
V*_ML_*	Velocity of the CoP medial–lateral
V*_AP_*	Velocity of the CoP anterior–posterior
IQR	Interquartile range
