# Supplementary material for: Telerehabilitation and Face-to-Face Exergame Delivery Modalities to Improve Postural Control in Children with Cerebral Palsy: A Randomised Controlled Trial
Source: Med Sci (Basel). 2026 May 8;14(2):246. doi: 10.3390/medsci14020246 (PMC13214957; doi:10.3390/medsci14020246)
Supplement: Supplementary file 1 [file medsci-14-00246-s001.zip › medsci-42497131-Supp.pdf]

medical sciences

MDPI

Supplementary Materials: Telerehabilitation and face-to-face exergame delivery modalities to improve postural control in children with cerebral palsy: a randomised controlled trial

Valeska Gatica-Rojas and L. Eduardo Cofré Lizama

Supplementary Table S1. Secondary outcome measures during six postural tasks for both groups over time.

			Baseline	Mid-intervention		End Intervention	Follow-ups		
			Week 0	Week 2	Week 4	Week 6	Week 8	Week 10	p-Value [a]
	Task	Group	Median [IQR]	Median [IQR]	Median [IQR]	Median [IQR]	Median [IQR]	Median [IQR]	(Kendall's W)
SD$_{ML}$ (cm)	EO	FF	6.60 [6.10 - 9.80]	9.60 [5.30 - 12.30]	5.00 [4.90 - 6.90]	2.50 [2.20 - 2.80]	6.10 [4.50 - 7.00]	3.50 [3.00 - 6.30]	< 0.01 (0.525)
		TR	10.60 [6.60 - 13.40]	9.00 [4.60 - 15.90]	6.10 [5.50 - 6.70]	2.70 [2.10 - 3.40]	2.80 [2.30 - 3.70]	4.10 [3.10 - 4.90]	< 0.01 (0.519)
	EC	FF	7.30 [5.10 - 8.30]	12.50 [7.60 - 14.10]	8.90 [5.10 - 16.20]	3.70 [2.90 - 5.80]	3.20 [2.40 - 5.70]	5.40 [3.20 - 6.20]	< 0.01 (0.691)
		TR	8.50 [6.80 - 10.10]	6.30 [5.50 - 8.10]	5.10 [3.50 - 7.00]	3.80 [2.40 - 4.70]	3.40 [3.10 - 4.30]	4.20 [2.90 - 6.10]	< 0.01 (0.605)
	ML-EO30	FF	76.50 [61.80 - 85.50]	66.70 [60.10 - 76.30]	70.90 [59.90 - 81.90]	53.70 [48.40 - 59.20]	48.60 [41.20 - 49.50]	53.40 [41.10 - 67.80]	< 0.01 (0.525)
		TR	80.40 [68.50 - 84.60]	74.20 [53.10 - 80.70]	69.90 [58.30 - 92.50]	57.20 [48.60 - 69.90]	51.70 [46.40 - 67.40]	67.40 [41.50 - 81.80]	0.22 (0.177)
	ML-EC30	FF	64.90 [62.20 - 79.40]	60.30 [49.90 - 74.70]	65.40 [52.60 - 83.40]	41.00 [38.30 - 55.10]	45.60 [43.60 - 49.20]	53.50 [42.40 - 56.80]	< 0.01 (0.593)
		TR	71.10 [63.00 - 86.30]	63.80 [56.50 - 69.70]	64.10 [56.90 - 78.20]	51.70 [36.90 - 60.40]	53.70 [49.50 - 72.90]	65.90 [43.70 - 68.70]	0.05 (0.275)
	ML-WS	FF	73.30 [70.30 - 82.00]	82.90 [76.50 - 87.60]	90.20 [89.10 - 92.20]	84.50 [70.70 - 90.70]	84.00 [78.60 - 92.40]	72.70 [71.70 - 92.00]	0.07 (0.291)
		TR	89.70 [68.50 - 97.00]	74.70 [62.70 - 93.70]	92.70 [75.80 - 102.70]	70.10 [52.40 - 90.00]	75.30 [66.30 - 88.00]	69.60 [63.60 - 85.40]	0.22 (0.175)
	AP-WS	FF	29.90 [24.90 - 31.30]	35.50 [26.70 - 37.10]	35.30 [32.30 - 37.80]	37.10 [30.50 - 39.50]	34.30 [30.10 - 44.20]	34.70 [30.10 - 40.50]	0.39 (0.149)
		TR	35.50 [33.20 - 49.90]	34.90 [24.50 - 48.00]	29.50 [27.70 - 36.50]	15.30 [14.20 - 26.20]	26.70 [25.40 - 30.40]	26.10 [25.10 - 27.40]	0.02 (0.347)
S	EO	FF	6.60 [5.10 - 7.80]	6.70 [4.90 - 8.20]	5.30 [4.60 - 7.20]	3.60 [3.10 - 4.30]	5.60 [5.30 - 6.50]	4.30 [3.60 - 7.50]	< 0.01
		TR	7.40 [6.40 - 9.80]	7.30 [6.40 - 9.40]	7.10 [6.10 - 8.20]	4.20 [4.00 - 5.10]	4.20 [3.30 - 6.20]	4.70 [4.20 - 5.40]	< 0.01 (0.4)
	EC	FF	7.10 [5.40 - 8.00]	6.50 [6.30 - 8.50]	7.50 [5.30 - 8.70]	4.90 [2.60 - 6.30]	5.60 [4.10 - 6.70]	5.60 [4.60 - 7.70]	0.07 (0.291)
		TR	8.70 [7.00 - 11.50]	9.20 [7.30 - 10.30]	9.20 [7.40 - 9.80]	5.80 [4.30 - 7.00]	5.90 [3.70 - 9.20]	6.40 [4.10 - 7.40]	< 0.01 (0.605)
	ML-EO30	FF	12.60 [10.90 - 12.80]	11.00 [10.00 - 12.70]	13.70 [11.90 - 15.40]	8.10 [6.50 - 12.00]	7.50 [6.10 - 9.90]	9.20 [8.10 - 10.70]	< 0.01 (0.459)
		TR	15.90 [12.10 - 19.10]	12.80 [11.70 - 15.60]	12.70 [9.90 - 15.20]	8.50 [7.80 - 11.80]	9.80 [8.30 - 11.40]	10.70 [9.30 - 11.60]	< 0.01 (0.519)
	ML-EC30	FF	12.50 [10.50 - 14.00]	12.20 [10.30 - 17.40]	13.20 [11.90 - 15.00]	9.20 [8.90 - 9.60]	11.00 [9.50 - 11.40]	10.20 [8.00 - 12.20]	< 0.01 (0.586)
		TR	15.70 [14.90 - 17.90]	14.70 [10.50 - 15.40]	14.30 [10.70 - 15.30]	10.70 [9.00 - 12.40]	10.50 [8.90 - 11.90]	9.90 [9.30 - 13.40]	< 0.01 (0.605)
	ML-WS	FF	16.20 [13.40 - 18.30]	20.30 [12.20 - 21.60]	14.00 [12.30 - 20.20]	13.50 [13.20 - 14.00]	14.50 [13.10 - 17.50]	14.20 [13.00 - 15.10]	0.89 (0.048)
		TR	16.20 [14.60 - 19.10]	15.70 [13.50 - 18.70]	16.80 [14.50 - 22.50]	12.60 [11.60 - 16.60]	12.80 [12.30 - 15.10]	15.50 [12.00 - 17.00]	0.11 (0.227)

 https://doi.org/10.3390/medsci14020246

	Task	Group	Baseline Week 0 Median [IQR]	Mid-intervention Week 2 Median [IQR]	Week 4 Median [IQR]	End Intervention Week 6 Median [IQR]	Follow-ups Week 8 Median [IQR]	Week 10 Median [IQR]	*p*-Value [a] (Kendall's W)
	**AP-WS**	**FF**	35.10 [30.40 - 42.50]	35.40 [26.00 - 40.30]	36.20 [31.70 - 43.30]	26.70 [16.00 - 32.00]	21.70 [18.90 - 32.60]	26.80 [22.10 - 32.40]	0.10 (0.263)
		**TR**	29.60 [23.10 - 41.40]	26.50 [22.90 - 37.20]	40.60 [35.80 - 43.70]	28.60 [26.70 - 39.90]	22.40 [16.80 - 33.30]	19.40 [17.80 - 31.20]	0.06 (0.266)
**V_{ML} (cm/s)**	**EO**	**FF**	14.10 [11.70 - 18.70]	16.20 [14.10 - 20.90]	15.20 [12.10 - 18.80]	8.80 [7.40 - 12.20]	13.50 [10.70 - 16.90]	11.20 [8.70 - 15.10]	0.02 (0.382)
		**TR**	13.30 [12.00 - 15.90]	13.20 [10.80 - 18.60]	13.40 [10.10 - 15.10]	8.30 [7.00 - 10.70]	9.00 [7.20 - 11.20]	10.80 [8.20 - 14.10]	< 0.01 (0.553)
	**EC**	**FF**	12.70 [10.90 - 18.00]	23.70 [13.50 - 26.50]	17.30 [13.90 - 27.30]	13.00 [7.90 - 14.80]	13.10 [8.20 - 15.50]	11.70 [10.10 - 13.80]	< 0.01 (0.601)
		**TR**	15.00 [11.60 - 17.20]	15.10 [11.70 - 18.00]	11.50 [11.10 - 20.50]	10.20 [7.40 - 11.60]	9.00 [8.00 - 14.10]	10.10 [8.80 - 12.20]	< 0.01 (0.565)
	**ML-EO30**	**FF**	116.80 [98.80 - 125.40]	104.00 [88.50 - 112.30]	109.70 [87.00 - 123.90]	78.70 [74.90 - 92.50]	74.10 [62.30 - 90.10]	91.70 [60.50 - 99.40]	0.01 (0.435)
		**TR**	118.70 [111.80 - 141.80]	106.40 [82.60 - 124.90]	110.80 [93.40 - 137.80]	89.20 [82.40 - 97.40]	91.30 [77.00 - 100.40]	108.30 [64.00 - 116.00]	0.05 (0.277)
	**ML-EC30**	**FF**	102.60 [99.30 - 109.80]	86.70 [80.50 - 118.70]	101.70 [83.30 - 115.20]	66.20 [59.00 - 91.50]	79.50 [65.20 - 94.00]	87.10 [66.00 - 93.20]	0.01 (0.45)
		**TR**	115.20 [97.90 - 136.00]	111.00 [86.50 - 119.30]	104.50 [93.50 - 138.90]	81.80 [65.10 - 97.40]	97.50 [75.20 - 105.10]	97.00 [72.20 - 106.60]	0.02 (0.348)
	**ML-WS**	**FF**	138.50 [123.90 - 217.60]	190.50 [152.90 - 205.70]	173.40 [164.30 - 192.70]	165.90 [139.20 - 179.60]	151.00 [145.60 - 187.70]	160.30 [137.40 - 174.30]	0.11 (0.258)
		**TR**	157.90 [131.00 - 203.00]	142.20 [126.10 - 167.80]	183.50 [133.00 - 214.50]	137.40 [119.10 - 152.60]	134.50 [116.60 - 153.90]	134.20 [118.80 - 157.90]	0.04 (0.286)
	**AP-WS**	**FF**	79.80 [62.90 - 107.40]	87.60 [71.30 - 106.20]	98.40 [92.60 - 119.40]	93.90 [71.40 - 97.10]	89.80 [74.40 - 92.90]	85.30 [81.90 - 95.00]	0.13 (0.244)
		**TR**	91.60 [84.80 - 111.20]	80.30 [69.20 - 118.30]	87.30 [74.50 - 103.40]	50.30 [42.80 - 58.00]	64.90 [51.80 - 80.60]	57.40 [52.60 - 67.90]	0.01 (0.388)
**V_{AP} (cm/s)**	**EO**	**FF**	12.60 [10.10 - 16.00]	16.10 [12.90 - 17.00]	13.80 [12.00 - 15.80]	9.00 [8.40 - 12.10]	11.10 [9.60 - 14.50]	10.70 [8.60 - 13.80]	0.03 (0.361)
		**TR**	12.60 [10.50 - 14.90]	12.70 [11.30 - 14.40]	11.30 [9.90 - 14.60]	9.60 [8.10 - 10.90]	10.00 [7.40 - 11.60]	9.80 [8.60 - 12.90]	< 0.01 (0.462)
	**EC**	**FF**	14.50 [11.00 - 17.40]	19.10 [15.60 - 20.70]	18.50 [15.40 - 20.90]	13.40 [8.00 - 14.70]	15.90 [9.20 - 17.10]	13.10 [11.40 - 15.60]	< 0.01 (0.61)
		**TR**	14.90 [12.00 - 20.30]	15.60 [13.60 - 18.70]	14.30 [11.70 - 16.50]	10.40 [9.20 - 14.70]	10.10 [9.00 - 16.20]	11.60 [8.80 - 14.20]	< 0.01 (0.582)
	**ML-EO30**	**FF**	29.70 [25.50 - 31.80]	25.40 [20.00 - 31.90]	30.10 [27.10 - 30.90]	21.90 [19.20 - 25.50]	20.70 [16.90 - 23.40]	20.70 [16.50 - 27.10]	0.01 (0.428)
		**TR**	32.80 [29.00 - 35.60]	29.30 [24.70 - 34.00]	27.20 [23.10 - 33.50]	21.10 [18.70 - 23.20]	21.60 [17.20 - 26.70]	25.70 [21.90 - 29.40]	0.02 (0.339)
	**ML-EC30**	**FF**	27.20 [26.30 - 32.70]	29.10 [27.80 - 37.90]	32.70 [30.50 - 39.20]	23.30 [20.50 - 28.60]	26.60 [19.50 - 31.70]	20.50 [19.00 - 32.20]	0.04 (0.34)
		**TR**	39.20 [35.70 - 45.60]	29.70 [22.50 - 44.10]	31.80 [25.50 - 40.00]	25.30 [19.70 - 28.00]	25.10 [18.80 - 29.30]	23.70 [22.20 - 32.50]	< 0.01 (0.426)
	**ML-WS**	**FF**	51.30 [41.60 - 66.80]	71.50 [42.60 - 86.70]	56.70 [49.40 - 75.60]	51.90 [44.20 - 57.30]	50.30 [41.90 - 68.60]	54.00 [41.60 - 68.20]	0.36 (0.156)
		**TR**	60.80 [52.40 - 69.80]	58.00 [46.60 - 68.70]	57.00 [49.20 - 66.00]	46.60 [36.70 - 50.40]	48.60 [44.80 - 52.40]	49.20 [43.10 - 53.00]	0.02 (0.339)
	**AP-WS**	**FF**	112.30 [79.10 - 132.90]	99.30 [68.10 - 136.30]	118.00 [99.20 - 158.60]	98.90 [51.20 - 113.30]	75.30 [56.70 - 91.60]	81.00 [67.90 - 95.30]	0.05 (0.319)
		**TR**	92.10 [73.00 - 111.50]	72.40 [57.80 - 113.70]	99.50 [82.40 - 106.20]	72.80 [63.80 - 86.20]	58.00 [41.40 - 73.20]	48.70 [46.00 - 71.10]	0.0378 (0.295)

[a] Friedman's one-way ANOVA. IQR: interquartile range. Measure, CoP: centre-of-pressure; SD_{ML} and SD_{AP}: standard deviation of CoP in the directions medial-lateral and anterior–posterior; V_{ML} and V_{AP}: CoP velocity in the directions medial-lateral and anterior–posterior. cm: centimetres; cm/s: centimetres/seconds. Task, EO: eyes open; EC: eyes closed; ML-EO30: Mediolateral weight shifting with EO at 30 bpm; ML-EC30: Mediolateral weight shifting with EC at 30 bpm;

ML-WS: mediolateral weight-shifting while playing Penguin; AP-WS: anteroposterior weight-shifting while playing Snowboard. Group, FF: face to face and TR: telerehabilitation.

Supplementary Table S2. Secondary clinical outcome measures during two clinical tests for both groups over time.

		Baseline	Mid-intervention		End Intervention	Follow-ups		
		Week 0	Week 2	Week 4	Week 6	Week 8	Week 10	
Test	Group	Median [IQR]	Median [IQR]	Median [IQR]	Median [IQR]	Median [IQR]	Median [IQR]	p-Value [a]
**MMAS-R**	**FF**	3.00 [2.50 - 4.00]	2.00 [1.50 - 3.00]	2.00 [1.00 - 2.50]	1.00 [0.50 - 1.50]	1.00 [0.50 - 1.50]	1.00 [0.50 - 1.50]	< 0.01
**(score)**	**TR**	3.00 [2.50 - 3.00]	2.00 [1.50 - 3.25]	2.00 [1.25 - 2.25]	2.00 [1.25 - 3.00]	1.00 [0.75 - 2.00]	1.00 [0.50 - 2.00]	0.09
**MMAS-L**	**FF**	2.00 [1.75 - 2.50]	2.00 [1.75 - 2.50]	1.00 [0.75 - 1.50]	1.00 [0.75 - 1.75]	1.00 [0.75 – 1.50]	1.00 [0.75 – 1.50]	0.42
**(score)**	**TR**	2.00 [1.75 - 2.50]	2.00 [1.50 - 2.50]	2.00 [1.70 - 2.50]	2.00 [1.70 - 2.50]	2.00 [1.70 - 2.50]	1.00 [1.70 - 2.50]	0.42
**TUG**	**FF**	7.52 [7.04 – 7.82]	7.85 [6.74 – 8.50]	6.83 [6.21 – 8.25]	6.55 [6.47 – 7.38]	6.41 [6.18 – 7.62]	6.82 [6.40 – 7.15]	0.02
**(s)**	**TR**	6.32 [5.81 – 7.01]	5.83 [5.51 – 6.64]	6.17 [5.55 – 6.63]	5.50 [5.00 – 5.67]	5.71 [5.03 – 6.00]	5.01 [4.80 – 6.13]	< 0.01

[a] Friedman's one-way ANOVA. IQR: interquartile range. MMAS: Modified Modified Ashworth Scale. TUG: Timed Up and Go. R: right; L: left; s: seconds. Group, FF: face to face and TR: telerehabilitation.
